# 

**Published:** 1904-02

**Authors:** 


### CONTENTS.

ORIGINAL COMMUNICATIONS—

INAUGURAL PRESIDENTIAL ADDRESS TO THE ATLANTA SOCIETY OF MEDICINE. By Alex W. Stirling, M.D., C.M. (Edin.); D. P. H. (Lond.), Atlanta, Ga. .... 721
SOME ADVANTAGES OF STAPLER'S RAREFIER IN THE TREATMENT OF THE EAR. By Maury M. Stapler, M.D., Macon, Ga. .... 730
A CASE OF POISONING BY HYOSCIN HYDROBROMID .... 741

SOCIETY REPORTS—

THE ATLANTA SOCIETY OF MEDICINE .... 742

CORRESPONDENCE—

MUST WE CURTAIL OUR WORK? .... 751

EDITORIAL NOTES AND COMMENTS—

RADIUM'S PLACE IN MEDICINE .... 753
ATLANTA SOCIETY OF MEDICINE .... 754
EDITORIAL BREVITIES .... 757

NEWS AND NOTES .... 758

CONTENTS CONTINUED .... x

